# Diseases of the Nose and Throat

**Published:** 1904-01

**Authors:** 


Diseases of the Nose and Throat. By Charles Huntoon Knight, A. M., M. D., Professor of Laryngology, Cornell University Medical College. Octavo, 438 pages; 147 illustrations. Philadelphia: P. Blakiston's Son & Co. 1903. (Price, $3.00.)

The volume described above is so uniformly written that no one part of it can be praised in preference to another. The author describes each disease and its treatment completely; states special facts concisely, and reviews the new theories with an unprejudiced pen. No better example of the concise and exhaustive manner in which he treats his subjects can be given, than his description of bony cysts of the middle turbinated body on the forty-fourth and succeeding pages. This book is thoroughly up to date and is especially planned for students as well as for general practitioners who wish to learn what is known on any subject pertaining to the specialty of diseases of the nose and throat. The reviewer has read the book with great pleasure and can think of nothing which could be added to make it more thoroughly complete. The illustrations are taken from recent sources and are of great assistance to a thorough understanding of the text.

